# Supplementary material for: A set of multi-entry identification keys to African frugivorous flies (Diptera, Tephritidae)
Source: Zookeys. 2014 Jul 24;(428):97–108. doi: 10.3897/zookeys.428.7366 (PMC4143993; doi:10.3897/zookeys.428.7366)
Supplement: Supplementary material 6 — Key to Ceratitis [file zookeys-428-097-s006.zip › SF6_ZooKeys_key to Ceratitis/key/SF6_ZooKeys_key to Ceratitis/Media/Html/Ceratitis whitei.htm]

Ceratitis (Pterandrus) whitei, n


***Ceratitis*** **(*Pterandrus*) *whitei* De Meyer & Freidberg**

Body
length: 4.45 (3.95-5.00) mm; wing length: 4.85 (4.20-5.30) mm.

Male

Head: Antenna yellow. First flagellomere three times as long
as pedicel. Arista with short to moderately long rays; ventral rays shorter and
sparser than dorsal rays, especially basally. Frons convex; pale, center more
yellow; with short scattered setulae largely of same color as frons. Face
yellowish white. Genal seta and setulae dark reddish or black, partly
developed.

Thorax: Postpronotal
lobe yellowish white, without spot, at most darker yellowish brown coloration
near postpronotal seta. Scutal pattern: ground color orange-brown, with streaks
and darker markings but without distinct spots or clearly defined stripes,
except prescutellar white markings, which are separate but with pale area in
between; microtrichia silvery or more golden colored. Scapular setae black.
Scutellum yellowish white, basally with two separate dark spots (not clear in
all specimens but this might be because of discoloration), apically with three
separate black spots, extending to basal 0.33. Anepisternum pale with ventral
half yellow (in some specimen completely yellow, perhaps also discoloration);
pilosity pale.

Legs:
Yellow except where otherwise noted; setation typical for subgenus, mixed pale
and dark. Foreleg: coxa brown; femur anteriorly brownish black; with short
black setulae, along ventral part with row of longer black setulae; posteriorly
with dorsal margin darker brown, with bush of longer setulae along entire
length, posterodorsal row longer and dark, others pale; ventral setae dark.
Midleg: femur anteriorly with short pale setulae, basally with few dark
setulae, no feathering; tibia dilated on ventral 0.66; with black feathering
dorsally along distal half and ventrally slightly less so; feathered part dark
colored. Hindleg: femur at apical 0.25 with longer pale setulae dorsally and
dark setulae ventrally.

Wing:
banding yellowish brown. Marginal band forming continuous band with anterior
part of discal band or at most incomplete one; cubital band free; medial band
absent; crossvein R-M at or just beyond middle of discal cell. Apex of vein R_1_
distal to level of crossvein R-M. Crossvein DM-Cu oblique anterobasally.

Abdomen:
Mostly yellow. Tergites 2 and 4 with pale gray band on posterior half. Tergite
3 with weak brown transverse band along� posterior 0.33. Male epandrium in
lateral view with posterior lobe of lateral surstylus straight and short,
apical end shortly curved.

Female

As
male except for the following characters: Genal seta and setulae all black,
well developed; setulae on frons darker than frons. Legs without feathering;
mainly yellow; forefemur with ventral setae and posterodorsal setulae dark;
posteroventral setulae pale. Oviscape shorter than preabdomen. Aculeus about
6-7 times longer than wide; tip with shoulder and lateral margin slightly
sinuous.

(Description
after De Meyer & Freidberg, 2006)
